# Disparities in Use of Novel Diabetes Medications by Insurance: A Nationally Representative Cohort Study

**DOI:** 10.1007/s11606-024-08961-x

**Published:** 2024-07-31

**Authors:** Lurit Bepo, Oanh K. Nguyen, Anil N. Makam

**Affiliations:** 1 Division of Hospital Medicine, San Francisco General Hospital, Department of Medicine, University of California, San Francisco, San Francisco, CA USA; 2 UCSF National Clinician Scholars Program, Philip R. Lee Institute for Health Policy Studies, University of California, San Francisco, San Francisco, CA USA; 3 UCSF Philip R. Lee Institute for Health Policy Studies, San Francisco, CA USA; 4 UCSF Center for Vulnerable Populations, San Francisco, CA USA; 5 Division of Hospital Medicine, Zuckerberg San Francisco General Hospital, Department of Medicine, University of California, San Francisco, CA USA

## Abstract

**Background:**

Minority racial and ethnic populations have the highest prevalence of type 2 diabetes mellitus but lower use of sodium-glucose co-transporter-2 inhibitors (SGLT2i) and glucagon-like peptide-1 receptor agonists (GLP1ra), novel medications that reduce morbidity and mortality. Observed disparities may be due to differences in insurance coverage, which have variable cost-sharing, prior authorization, and formulary restrictions that influence medication access.

**Objective:**

To assess whether racial/ethnic differences in SGLT2i and GLP1ra use differ by payer.

**Design:**

Cross-sectional analysis of 2018 and 2019 Medical Expenditure Panel Survey data.

**Participants:**

Adults ≥ 18 years old with diabetes.

**Main Measures:**

We defined insurance as private, Medicare, or Medicaid using ≥ 7 months of coverage in the calendar year. We defined race/ethnicity as White (non-Hispanic) vs non-White (including Hispanic). The primary outcome was use of ≥ 1 SGLT2i or GLP1ra medication. We used multivariable logistic regression to assess the interaction between payer and race/ethnicity adjusted for cardiovascular, socioeconomic, and healthcare access factors.

**Key Results:**

We included 4997 adults, representing 24.8 million US adults annually with diabetes (mean age 63.6 years, 48.8% female, 38.8% non-White; 33.5% private insurance, 56.8% Medicare, 9.8% Medicaid). In our fully adjusted model, White individuals with private insurance had significantly more medication use versus non-White individuals (16.1% vs 8.3%, *p* < 0.001), which was similar for Medicare beneficiaries but more attenuated (14.7% vs 11.0%, *p* = 0.04). Medication rates were similar among Medicaid beneficiaries (10.0% vs 9.0%, *p* = 0.74).

**Conclusions:**

Racial/ethnic disparities in novel diabetes medications were the largest among those with private insurance. There was no disparity among Medicaid enrollees, but overall prescription rates were the lowest. Given that disparities vary considerably by payer, differences in insurance coverage may account for the observed disparities in SGLT2i and GLP1ra use. Future studies are needed to assess racial/ethnic differences in novel diabetes use by insurance formulary restrictions and out-of-pocket cost-sharing.

**Supplementary Information:**

The online version contains supplementary material available at 10.1007/s11606-024-08961-x.

## BACKGROUND

Minority racial and ethnic populations have disproportionately higher prevalence of type 2 diabetes mellitus and associated complications compared to White individuals.^1–3^ Improving uptake of evidence-based pharmacotherapy could narrow the equity gap by decreasing diabetes-related morbidity and mortality. Sodium-glucose co-transporter-2 inhibitors (SGLT2i) and glucagon-like peptide-1 receptor agonists (GLP1ra) are novel classes of medications for type 2 diabetes that significantly reduce cardiovascular deaths independent of their effect on hyperglycemia and are widely endorsed by clinical practice guidelines.^4–11^ Although > 80% of patients with type 2 diabetes are eligible for these medications due to the presence of cardiovascular or renal risk factors, only 1 in 10 patients receives either an SGLT2i or GLP1ra medication.^12,13^ Compounding this significant underuse, Black, Hispanic/Latinx, and Asian individuals receive fewer prescriptions for these medications compared to White individuals.^14–17^ Failing to address racial/ethnic disparities in use of these medications will exacerbate already existing disparities in type 2 diabetes mellitus and associated cardiovascular outcomes.^1,3^

One potential but unexplored reason for the racial/ethnic disparities in SGLT2i and GLP1ra medication use is insurance coverage. As with other patented drugs, these novel medications are expensive with median retail prices ranging from $300 to $942 per month.^18–20^ Thus, insurance plans may implement policies to restrict medication access and contain costs, including patient cost-sharing and formulary restrictions, prior authorization, and step therapy requirements. Previous studies have demonstrated that patients insured by plans with more formulary restrictions and higher out-of-pocket expenses for certain medications receive significantly fewer prescriptions of those medications.^18,19,21,22^ However, no prior studies have specifically assessed the association between insurance and racial/ethnic disparities in SGLT2i and/or GLP1ra use. This is important because if insurance coverage is significantly associated with racial/ethnic disparities in access to lifesaving diabetes medications, then removing formulary restrictions and minimizing cost-sharing for these medications across all payers would be important policy levers that could meaningfully improve health equity in diabetes.

Therefore, we conducted a nationally representative cohort study to assess the extent to which race/ethnicity, insurance, and their interaction are associated with SGLT2i and GLP1ra medication use.

## METHODS

### Study Design, Population, and Data Sources

We conducted an observational cohort study via retrospective analysis of publicly available survey data from the Medical Expenditure Panel Survey (MEPS), focusing on adults ≥ 18 years old with self-reported diabetes mellitus (DM). MEPS is a nationally representative database of large-scale surveys of US civilian, noninstitutionalized families and individuals, their medical providers, and their employers sponsored by the Agency for Healthcare Research and Quality. Participants are drawn from a subsample of households who participated in the National Health Interview Survey conducted by the National Center for Health Statistics (NCHS) and provide data on the cost and use of healthcare and on health insurance coverage across the USA.

MEPS gathers data from a panel of participants over five rounds of interviews spanning two calendar years. Data from a single calendar year therefore consists of multiple rounds collected from different panels. For our study, we merged data from the MEPS Household Component (MEPS-HC) and Prescribed Medications files for calendar years 2018 and 2019, representing two full calendar years of data. The presence of DM was defined as an affirmative response to the MEPS survey question of whether the participant had ever been diagnosed with diabetes, excluding gestational diabetes. Because of limited sample size, we excluded participants who were uninsured or had insurance coverage other than private, Medicare, or Medicaid, including Veterans Affairs and non-employer group plans.

### Primary Exposure

Our primary exposure of interest was the interaction between race/ethnicity and insurance. We defined race/ethnicity using self-reported categories from MEPS that allowed participants to indicate mutually exclusive racial and ethnic combinations (i.e., Hispanic, non-Hispanic White, non-Hispanic Black, non-Hispanic Asian, and non-Hispanic other race/multi-race; hence our reference throughout to “race/ethnicity” rather than “race and ethnicity”). In multivariable analyses, due to small sample sizes for disaggregated racial/ethnic groups for our analyses, we compared non-Hispanic White (herein referred to as “White”) to all other racial and ethnic groups (herein referred to as “non-White”).

We ascertained insurance from monthly health insurance indicators and defined mutually exclusive categories of private insurance, Medicare, or Medicaid based on participant reporting of ≥ 7 months of each respective insurance within the calendar year, as has been done in prior MEPS studies.^23^ For individuals with multiple payers, we used the hierarchy of Medicare > private insurance > Medicaid. Thus, individuals with Medicare and either private insurance or Medicaid were categorized as having Medicare since prescription drug coverage for both these groups is primarily covered through Medicare Part D rather than through private Medigap supplemental coverage or Medicaid.

### Primary Outcome

Our primary outcome was the receipt of ≥ 1 SGLT2i or GLP1ra prescribed medication in a calendar year using the Multum Lexicon specific codes 458 and 373, respectively, from the MEPS Prescribed Medicines files. Obtaining a prescribed medication was based on household report and corroborated using pharmacy data for participants who provided written consent.

### Measures of Cardiovascular Risk

We assessed cardiovascular risk using age, sex, and self-reported prior diagnoses of coronary artery disease (CAD; which we defined as any “coronary heart disease,” “angina pectoris,” or “myocardial infarction”), “high cholesterol,” and “high blood pressure” which were available within the MEPS-HC file. Prior CAD was endorsed by the ADA guidelines on managing diabetes as an indication for SGLT2i or GLP1ra in 2018.^24^ Cardiovascular risk factors in the absence of CAD were also included as inclusion criteria in several key landmark trials published during our study period.^25^

### Measures of Socioeconomic Status and Healthcare Access

We included self-reported education, marital status, family income, presence of a usual source of care, and enrollment in a managed care plan as covariates to assess socioeconomic status and healthcare access. Educational attainment was based on the highest degree obtained when a participant first entered MEPS, which we classified as high school/GED or greater. Current marital status was assessed during each interview round, which we classified as married versus not married. MEPS defines family income as the sum of annual earnings for all MEPS-reported family members using the 2018 poverty statistics developed by the Current Population Survey (CPS). MEPS also computes family income as a percent of the federal poverty level by dividing total family income by the federal poverty level (FPL) corresponding to each participant’s CPS family size and composition. We used this percent (%FPL) in our statistical analysis to standardize income by household size and composition. Usual source of care is defined by MEPS as “a particular doctor’s office, clinic, health center, or other place where [participants go] when they [are] sick or [need] medical advice” (MEPS HC-209 2018 Full Year Consolidated Data File, August 2020; MEPS HC-216 2019 Full Year Consolidated Data File, August 2021). Lastly, we defined whether participants were enrolled in a managed care plan using indicators in MEPS because these plan types may influence prescriptions via more restrictive formulary restrictions or greater cost-sharing.

### Statistical Analysis

#### Survey Weighting

We accounted for the complex survey design in all analyses as per MEPS guidance for data use.^26,27^ We averaged the survey weights over 2018 and 2019 such that all weighted results reflect the average annual population size for the pooled time period (MEPS HC-036: 1996–2020 Pooled Linkage Variance Estimation File). We determined the raw unweighted sample size to characterize the cohort and ascertain prevalence of medication use and applied survey weights to obtain nationally representative estimates.

#### Sequential Modeling

SGLT2i and GLP1ra medications are indicated for patients with type 2 diabetes with CV risk factors, but their use may also be affected by socioeconomic and healthcare access factors. To investigate the effect of race/ethnicity and insurance on the receipt of these medications, we conducted a three-part sequential logistic regression analysis to assess how these different factors may modulate the effect, with our primary focus being the fully adjusted model. The three models included (1) a base model consisting of race/ethnicity, insurance, and the interaction between these two covariates; (2) the base model plus the CV risk factors defined above to assess whether disparities widen given that non-White individuals may have more CV risk factors; and (3) the fully adjusted model, which additionally adjusted for the socioeconomic and access to care factors defined above that may mediate the effect of race/ethnicity and therefore attenuate disparities in medication use. For all models, we imputed missing data for binary variables as not present, which was minimal for included covariates (ranging from 0 to 1.6%).

For model development, we assessed normality and linearity assumptions for our continuous age and family income variables, and we transformed age into a cubic-spline version to account for violation of these assumptions. Analyses for model diagnostics revealed adequate fit and were not affected by influential or outlier values. For each model, we presented the marginal predicted probability of medication use and the odds ratio for White versus non-White individuals for each insurance type; we considered a *p* value < 0.05 to be statistically significant. All analyses were conducted using STATA 17 (StataCorp).

This study was exempt from review per UCSF institutional review board guidelines as MEPS uses publicly available, de-identified data.

## RESULTS

There were 4997 adults with DM in the 2018 and 2019 MEPS surveys, representing an average annual estimate of approximately 24.8 million adults in the USA with diabetes. Table 1 shows the sociodemographic, cardiovascular (CV) risk, socioeconomic status (SES), and access to care characteristics for the study cohort by payer. Overall, the mean age was 63.6 years (SD ± 13.6), and 48.8% were female. One-third (33.5%) had private insurance, 56.8% had Medicare, and 9.8% had Medicaid. Those with private insurance had the highest education and income and lowest prevalence of CV risk factors. Individuals with Medicare were older and had greater CV risk. Those insured by Medicaid had more racial/ethnic diversity, as well as lower income and education. Usual source of care was high (> 80%) across all insurance types.

**Table 1:** Characteristics of Adults with Diabetes in 2018–2019 Medical Expenditure Panel Survey (*n* = 4997) (All Percentages Are Weighted to Account for Complex MEPS Survey Design)

Variable	All diabetes	Private insurance	Medicare	Medicaid
*N*	4997	1393 (33.5%)	3032 (56.8%)	572 (9.8%)
Weighted annual population	24,757,803	8,290,186	14,052,798	2,414,819
Non-White race/ethnicity	2327 (38.8%)	632 (39.2%)	1312 (34.7%)	383 (61.0%)
Black or African American	962 (14.4%)	225 (12.8%)	593 (14.1%)	144 (21.2%)
Hispanic or Latino	942 (15.0%)	262 (15.2%)	505 (13.2%)	175 (25.3%)
Asian	234 (5.8%)	80 (6.8%)	123 (4.7%)	31 (8.0%)
Other	189 (3.6%)	65 (4.4%)	91 (2.7%)	33 (6.5%)
Cardiovascular risk factors				
Age, mean in years (± SD)	63.6 (± 13.6)	52.8 (± 9.6)	71.3 (± 9.1)	48.7 (± 11.8)
Female sex	2589 (48.8%)	669 (44.1%)	1541 (49.2%)	379 (62.9%)
ASCVD, any	1116 (21.7%)	160 (11.8%)	866 (28.6%)	90 (15.9%)
Coronary heart disease	831 (16.4%)	109 (8.2%)	664 (22.7%)	58 (8.3%)
Angina	347 (7.1%)	59 (4.5%)	258 (8.9%)	30 (5.5%)
Myocardial infarction	627 (12.3%)	88 (6.5%)	493 (16.4%)	46 (8.2%)
Hypertension	3844 (75.2%)	918 (65.2%)	2547 (82.9%)	379 (64.2%)
High cholesterol	3639 (73.0%)	916 (65.6%)	2365 (79.2%)	358 (62.3%)
Socioeconomic status and healthcare access factors				
High school education/GED or less	3486 (65.6%)	790 (53.2%)	2210 (70.0%)	486 (82.5%)
Currently married	2597 (54.4%)	927 (65.8%)	1469 (51.1%)	201 (35.3%)
Family income, median, $ (IQR)*	41,764 (18,664–81,600)	77,000 (46,500–119,828)	33,872 (15,880–67,000)	18,343 (9000–35,142)
Family income as % of FPL, median (IQR)	243 (120–456)	38 (251–602)	21 (11–415)	96 (53–173)
Has usual source of care	4456 (89.1%)	1218 (86.5%)	2763 (91.8%)	475 (82.6%)
Managed care plan^†^	2474 (48.7%)	565 (39.9%)	1657 (54.6%)	252 (44%)
Medication use				
Any SGLT2i or GLP1ra	611 (12.9%)	237 (17.3%)	313 (10.9%)	61 (9.9%)
SGLT2i	264 (5.6%)	111 (8.1%)	127 (4.4%)	26 (4.3%)
GLP1ra	416 (8.7%)	161 (11.5%)	211 (7.4%)	44 (7.0%)

Abbreviations: *ASCVD*, atherosclerotic cardiovascular disease; *FPL*, federal poverty level; *GED*, general educational diploma; *GLP1ra*, glucagon-like peptide-1 receptor agonist; *IQR*, interquartile range; *SD*, standard deviation; *SGLT2i*, sodium-glucose co-transporter-2 inhibitor

^*^Computed variable in MEPS by dividing total family income by the FPL corresponding to each MEPS participant’s Center for Population Statistics-defined family size and composition

^†^Constructed variable in MEPS based on round- and calendar year-specific survey data

### Descriptive Findings of Medication Use

Overall, only about 1 in 8 individuals (12.9%) used any SGLT2i or GLP1ra medication. Individuals with private insurance had the highest use (17.3%) compared to Medicare (10.9%) and Medicaid (9.9%). White individuals had higher use (15.2%) than non-White individuals (9.4%), ranging from 8.9 to 10.1% for disaggregated race/ethnicity groups (Appendix Table 1). Among the subgroup of patients with prior CAD, there were large differences in medication use by payer (30.9% for private vs 11.2% for Medicare), but to a lesser extent by race/ethnicity (14.4% for White vs 13.6% for non-White individuals; Appendix Table 2). However, estimates were too imprecise to explore use by payer and disaggregated race/ethnicity due to small sample sizes.

### Medication Use from Sequential Models

**Figure 1.**
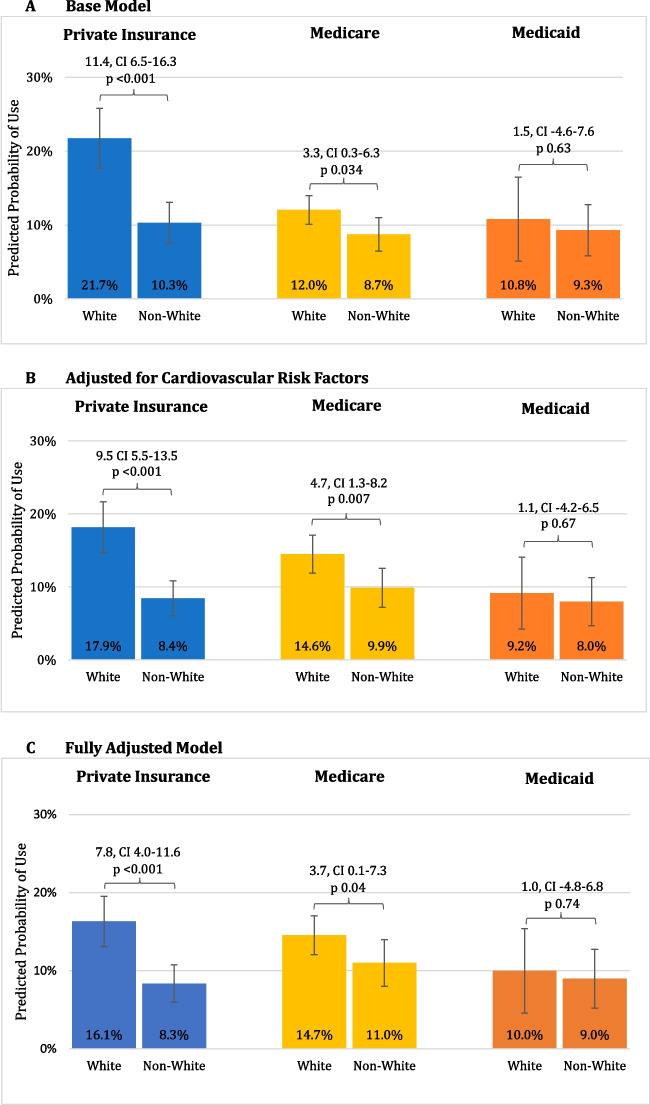
Predicted probabilities of any SGLT2i and GLP1ra medication use. Panel A: Base model. Panel B: Model adjusted for cardiovascular risk factors. Panel C: Fully adjusted model. Abbreviations: CI, 95% confidence interval; GLP1ra, glucagon-like peptide-1 receptor agonist; SGLT2i, sodium-glucose co-transporter-2 inhibitor. The three panels in this figure show the predicted probability of any SGLT2i and GLP1ra medication use in (**A**) the base model; (**B**) the base model adjusted for age, sex, and diagnosis of CAD, hypertension, and high cholesterol; and (**C**) the base + cardiovascular risk models further adjusted for education, family income as a percent of federal poverty level, marital status, presence of usual source of care, and managed care plan.

In our unadjusted base model, White individuals with private insurance and, to a lesser extent, Medicare had higher medication use of a novel diabetes medication compared to non-White individuals (21.7% vs 10.3%, *p* < 0.001 for private insurance; 12.0% vs 8.7%, *p* = 0.034 for Medicare) (Fig. 1A). There was no statistically significant difference between White and non-White individuals with Medicaid (10.8% vs 9.3%, *p* = 0.63). White individuals had an increased odds of SGLT2i or GLP1ra medication use compared to non-White individuals for private insurance (OR 2.42, 95% CI 1.65–3.53) and Medicare (OR 1.43, 95% CI 1.01–2.01) (Table 2).

**Table 2:** Unadjusted and Adjusted Odds Ratio of SGLT2i and GLP1ra Use by Race and Ethnicity and Insurance

	Base model*		**CV risk model**^**†**^		**Fully adjusted model**^**‡**^	
	**Odds ratio (95% CI)**	***p* value**	**Odds ratio (95% CI)**	***p* value**	**Odds ratio (95% CI)**	***p* value**
Private, non-White	Reference		Reference		Reference	
Private, White	2.42 (1.65–3.53)	< 0.001	2.42 (1.66–3.54)	< 0.001	2.17 (1.48–3.18)	< 0.001
Medicare, non-White	Reference		Reference		Reference	
Medicare, White	1.43 (1.01–2.01)	0.042	1.58 (1.12–2.22)	0.010	1.41 (0.99–1.99)	0.05
Medicaid, non-White	Reference		Reference		Reference	
Medicaid, White	1.18 (0.61–2.27)	0.62	1.16 (0.59–2.26)	0.67	1.13 (0.57–2.23)	0.73

Abbreviations: *CI*, confidence interval; *CV*, cardiovascular; *GLP1ra*, glucagon-like peptide-1 receptor agonist; *SGLT2i*, sodium-glucose co-transporter-2 inhibitor

^*^The base model includes race/ethnicity, insurance, and the interaction between these two covariates

^†^The CV risk model additionally adjusts for age, sex, and diagnosis of CAD, hypertension, and high cholesterol

^‡^The fully adjusted model additionally adjusts for education, family income as a percent of federal poverty level, marital status, presence of usual source of care, and managed care plan

After adjusting for cardiovascular risk factors, the disparity between White and non-White groups persisted for both private insurance and Medicare (Fig. 1B and Table 2). There was no statistically significant difference for Medicaid (9.2% vs 8.0%, *p* = 0.67; OR 1.16, 95% CI 0.59–2.26).

After additional adjustment for socioeconomic status and access to care, the White vs non-White disparity was still present but attenuated for private insurance (Fig. 1C and Table 2; full model available in Appendix Table 3). This was also true for Medicare to a lesser extent, but the difference was not statistically significant. There remained no statistically significant difference for Medicaid.

## DISCUSSION

In this nationally representative cohort study, we found considerable racial/ethnic disparities in the receipt of novel diabetes medications that were dependent on the type of insurance. White individuals with private insurance had more than double the medication use of non-White privately insured individuals. This disparity persisted after adjustment for cardiovascular risk, socioeconomic status, and access to care. A similar pattern of disparities, though much smaller in magnitude, was seen for Medicare beneficiaries. Although we found no significant differences in use among Medicaid enrollees by race/ethnicity, overall medication rates were the lowest versus individuals with private insurance or Medicare. However, we cannot exclude a possibility of either meaningfully lower or higher medication use among non-White Medicaid enrollees given wide confidence intervals. Because the magnitude of the disparity in medication use between White and non-White populations depended on the type of insurance, one potential key lever to improve health equity for individuals with diabetes is to address insurance plan policies that restrict access to novel diabetes medications, such as high out-of-pocket costs and formulary restrictions, to increase use of these highly effective medications for diabetes. Our findings may have even broader implications for pharmacoequity beyond diabetes given the expanding evidence base supporting the benefit of these medications in other conditions that affect large segments of the US population, including obesity, congestive heart failure, and chronic kidney disease.^28–31^

Consistent with our findings, other studies have demonstrated low rates of SGLT2i and GLP1ra medication use (11.2% and 8%, respectively), even among patients at high risk of cardiovascular disease.^12–16,32^ Recent studies have also broadly shown racial and ethnic differences in SGLT2i and GLP1ra prescriptions with Black, Hispanic/Latinx, and Asian groups receiving these medications at lower rates than White individuals.^14–17^ Our findings from this nationally representative sample extend the existing literature by showing that the extent of racial/ethnic disparities in receipt of novel diabetes medications depends on the type of insurance—worst for private insurance and potentially nonexistent for Medicaid.

Differences in medication formularies and cost-sharing are potential drivers of use and observed racial/ethnic disparities in SGLT2i and GLP1ra medications between payers.^18,21,33^ Medicare plans have the greatest coverage of SGLT2i medications without prior authorization or step therapy, whereas Medicaid plans have the most restrictions to access.^21^ Conversely, Medicaid plans consistently have the lowest out-of-pocket costs.^19,21–23,34^ Thus, our findings imply that formulary access restrictions may influence overall prescription rates (highest in private insurance and Medicare but lowest in Medicaid), but cost-sharing may be most culpable in driving racial/ethnic disparities. Namely, the low but equitable prescription rates in Medicaid may be due to restricted access and low cost-sharing, respectively. These findings have practical implications for policy solutions that target practices at the insurance plan level to reduce high out-of-pocket costs and remove formulary restrictions. In doing so, it may be possible to reduce disparities in SGLT2i and GLP1ra use to achieve pharmacoequity and ultimately decrease racial/ethnic disparities in diabetes health outcomes.^31^

There is also wide variability *within* insurance types in terms of cost-sharing and formulary restrictions, which we were unable to explore in this study.^18,22,33^ For example, with regard to cost-sharing, co-payments and co-insurance are far more variable among private insurance plans than Medicare, in contrast to the nearly uniformly low out-of-pocket costs for Medicaid plans.^21^ The persistent racial/ethnic disparity we observed for private insurance may be explained by lower quality of coverage among privately insured minoritized populations who may face higher out-of-pocket costs and more restrictive formularies.^35^ This might also account for why medication use among non-White individuals with private insurance approached those of the Medicaid enrollees in our study. Regarding formulary restrictions, two-thirds of fee-for-service Medicaid beneficiaries have unrestricted access to an SGLT2i or GLP1ra medication versus just one-third of managed care Medicaid enrollees, with significant variation in access both across and within states.^33^ Future research should explicitly study the link between out-of-pocket costs for patients, formulary restrictions, and receipt of an SGLT2i or GLP1ra medication versus a dipeptidyl peptidase-4 inhibitor as a negative control, which are also novel diabetes medications but lack cardiovascular or mortality benefit.

Another potential explanation for why we observed starkly different disparities by insurance type includes structural racism embedded in health systems, and implicit or explicit bias among the clinicians that may serve these different populations.^17,35–37^ However, the differential pattern by payer suggests that differences in insurance plan may be bigger drivers of disparities than health system- or clinician-level factors.

Our study had certain limitations. First, we were unable to further disaggregate race/ethnicity in adjusted analyses due to insufficient sample size in the MEPS cohort. However, our unadjusted analyses confirmed a similar pattern in the magnitude of racial/ethnic disparities being largest for private insurance and smallest for Medicaid. Second, our insurance categories illustrate broad patterns in medication prescriptions but are unable to capture the variability of plan formularies and cost-sharing implications within payers. Third, we were unable to distinguish between type 1 and type 2 diabetes mellitus from data available in MEPS. Given the low national prevalence of type 1 diabetes, this is unlikely to meaningfully affect our estimates. Additionally, there is emerging data that SGLT2is may also be beneficial in type 1 diabetes.^38,39^ Fourth, we lacked reliable data on additional comorbidities (i.e., heart failure, chronic kidney disease, or strokes), laboratory measures (i.e., glucose, hemoglobin A1c, creatinine, microalbuminuria), and medications (i.e., insulin) that signify greater cardiovascular risk or worse diabetes control. Finally, contemporary medication rates are likely higher than those found in our study period from 2018 to 2019 given increased awareness and stronger practice guideline recommendations for individuals at higher cardiovascular risk without established CAD. While our descriptive findings show narrower disparities for individuals with diabetes and prior CAD, it is possible that racial and ethnic disparities are still present despite higher overall medication use.

In conclusion, racial and ethnic disparities in novel diabetes medication use differ by insurance, with the greatest disparities observed among those with private insurance and which persist after adjusting for cardiovascular, socioeconomic status, and access to care factors. Disparities in medication use were the lowest in Medicaid and were not statistically significant; however, overall medication use was far lower among those with Medicaid than those with Medicare or private insurance. These findings suggest differences may be driven by different plan formulary and cost-sharing policies, which are potential policy levers to improve health equity.

## Supplementary Information

Below is the link to the electronic supplementary material.

Supplementary file1 (DOCX 25 KB)
