# Supplementary material for: Disparities in Use of Novel Diabetes Medications by Insurance: A Nationally Representative Cohort Study
Source: J Gen Intern Med. 2024 Jul 31;39(15):2987–94. doi: 10.1007/s11606-024-08961-x (PMC11576692; doi:10.1007/s11606-024-08961-x)
Supplement: Supplementary file 1 — Supplementary file1 (DOCX 25 KB) [file 11606_2024_8961_MOESM1_ESM.docx]

**ONLINE SUPPLEMENT**

**Disparities in Use of Novel Diabetes Medications by Insurance: A Nationally Representative Cohort Study**

Lurit Bepo, MD, MPH^1,2*^, Oanh K. Nguyen, MD, MAS^1, 3, 4^, Anil N. Makam, MD, MAS^1, 3, 4^

^1^Division of Hospital Medicine, San Francisco General Hospital, Department of Medicine, University of California, San Francisco, CA

^2^UCSF National Clinician Scholars Program, Philip R. Lee Institute for Health Policy Studies, University of California, San Francisco, San Francisco, CA

^3^ UCSF Philip R. Lee Institute for Health Policy Studies, San Francisco, CA

^4^ UCSF Center for Vulnerable Populations, San Francisco, CA

| Appendix Table 1. Medication Use by Race/Ethnicity and Insurance……………………………... | 2 |
| --- | --- |
| Appendix Table 2. Medication Use by Race/Ethnicity and Insurance Among Patients with CAD... | 2 |
| Appendix Table 3. Fully Adjusted Model for Medication Use.……………………………………. | 3 |

| **Appendix Table 1: Any SGLT2i or GLP1ra Medication Use by Race/Ethnicity and Insurance ^a^** | | | | |
| --- | --- | --- | --- | --- |
| **Race/ethnicity** | **All Payers** | **Private** | **Medicare** | **Medicaid** |
| White, non-Hispanic | 391/2670 (15.2%) | 170/761 (21.7%) | 200/1720 (12.0%) | 21/189 (10.8%) |
| Non-White | 220/2327 (9.4%) | 67/632 (10.3%) | 113/1312 (8.8%) | 40/383 (9.3%) |
| Black | 79/962 (8.9%) | 21/225 (8.4%) | 40/593 (8.1%) | --* |
| Hispanic or Latino | 101/942 (10.1%) | 32/262 (13.0%) | 51/505 (8.8%) | --* |
| Other | 40/423 (9.0%) | --* | 22/214 (9.8%) | --* |
| Total | 61/4997 (12.9%) | 237/1393 (17.3%) | 313/3032 (10.9%) | 61/572 (9.9%) |
| Abbreviations: SGLT2i, sodium glucose co-transporter-2 inhibitor. GLP1ra, glucagon-like peptide-1 receptor agonist.  ^a^ For each cell, n/N represents the observed number of individuals with medication use (n) over the eligible denominator for the respective race/ethnicity-payer permutation (N). All percentages are weighted to account for complex MEPS survey design.  * Estimate suppressed due to imprecision of estimates per MEPS guidance. | | | | |

| **Appendix Table 2: Any SGLT2i or GLP1ra Medication Use by Race/Ethnicity and Insurance**  **Among Patients with Prior CAD ^a^** | | | | |
| --- | --- | --- | --- | --- |
| **Race/ethnicity** | **All Payers** | **Private** | **Medicare** | **Medicaid** |
| White | 100/683 (14.4%) | 33/100 (30.9%) | 62/544 (11.2%) | --* |
| Non-White | 58/433 (13.6%) | --* | 36/322 (10.0%) | --* |
| Total | 158/1116 (14.1%) | 47/160 (28.6%) | 98/866 (10.9%) | --/90* |
| Abbreviations: SGLT2i = sodium glucose co-transporter-2 inhibitor. GLP1ra = glucagon-like peptide-1 receptor agonist. CAD = coronary artery disease.  ^a^ For each cell, n/N represents the observed number of individuals with medication use (n) over the eligible denominator for the respective race/ethnicity-payer permutation (N). All percentages are weighted to account for complex MEPS survey design.  * Estimate suppressed due to imprecision of estimates per MEPS guidance. | | | | |

| **Appendix Table 3: Fully Adjusted Model for Use of an SGLT2i or GLP1ra ^a^** | |
| --- | --- |
| **Race/ethnicity-Insurance** | **Adjusted Odds Ratio (95% CI)** |
| Private, Non-White | reference |
| Private, White | 2.17 (1.48-3.18) |
| Medicare, Non-White | reference |
| Medicare, White | 1.41 (0.99-1.99) |
| Medicaid, Non-White | reference |
| Medicaid, White | 1.13 (0.57-2.23) |
| **Cardiovascular Risks** |  |
| Age cubic spline term 1 ^b^ | reference |
| Age cubic spline term 2 ^b^, per year | 1.09 (1.04-1.16) |
| Age cubic spline term 3 ^b^, per year | 0.91 (0.87-0.95) |
| Female sex | 1.27 (1.02-1.58) |
| Prior coronary artery disease | 1.29 (0.97-1.71) |
| Hypertension history | 1.08 (0.82-1.41) |
| High cholesterol history | 1.24 (0.90-1.63) |
| **SES and Access to Care** |  |
| Greater than high school education/GED | 1.24 (0.97-1.60) |
| Married | 1.29 (1.01-1.66) |
| Family income, per 1% of FPL | 1.0001 (0.9998-1.0005) |
| Has a usual source of care | 1.61 (1.13-2.30) |
| Enrolled in a managed care plan | 0.64 (0.52-0.79) |
| Abbreviations: SGLT2i, sodium glucose co-transporter-2 inhibitor. GLP1ra, glucagon-like peptide-1 receptor agonist; SES, socioeconomic status; FPL, federal poverty limit.  ^a^ All findings are weighted to account for complex MEPS survey design.  ^b^ The three cubic spline knots for age were 24, 49, and 74 years old. | |
